# 한국 일개 종합병원의 병원발생 혈류감염의 역학적 특성 및 말초정맥카테터 관련 혈류감염의 위험요인 분석: 후향적 사례-대조군 연구

**DOI:** 10.7586/jkbns.25.003

**Published:** 2025-02-25

**Authors:** Chae Eun Kim, Soo Min Lee

**Affiliations:** College of Nursing, Dong-A University, Busan, Korea; 동아대학교 간호학과

**Keywords:** Catheter-related infections, Cross infection, Infection control, Bacteremia, 카테터관련 감염, 의료관련감염, 감염관리, 혈류감염

## Abstract

**Purpose:**

This study investigated the incidence of hospital-onset bloodstream infections (HOBSI) and risk factors for HOBSI among patients with peripheral venous catheters (PVCs) at a general hospital.

**Methods:**

A retrospective case-control study was conducted on patients hospitalized between August 1, 2021, and July 31, 2023. The case group included 31 patients diagnosed with bloodstream infections, while the control group included 93 patients without bloodstream infections. The control group were matched to the case group at a 1:3 ratio using propensity score matching. Data were analyzed using the chi-square test, t-test, and logistic regression analysis.

**Results:**

The incidence density per 1,000 patient-days was 0.61 for HOBSI-1, 0.40 for HOBSI-2, and 0.25 for central line-associated bloodstream infections (CLABSI). Approximately 70%-80% of bloodstream infections were found to occur in general wards. Multivariate analysis revealed that prolonged hospital stays (odds ratio [OR] =1.03) and liver cirrhosis (OR = 14.76) were significant risk factors for PVC-associated bloodstream infection (PVC-BSI). The mortality rate in the case group was significantly higher, with a 22.08-fold increased risk compared to the control group.

**Conclusion:**

This study revealed that HOBSI had a higher incidence rate than CLABSI, with the majority of cases occurring in general wards. HOBSI surveillance demonstrated relatively higher sensitivity, which can be beneficial for identifying bloodstream infections in hospitals with low catheter usage. Given the high mortality risk, PVC-BSI require increased attention. This study provides valuable data for improving bloodstream infection prevention strategies.

## 서론

### 1. 연구의 필요성

혈류감염은 의료관련감염 중 주 감염 부위가 혈류인 것으로 혈관카테터가 주요 위험요인으로 알려져 있다[1-3]. 혈류감염은 국내 의료관련감염 중 가장 빈번하게 발생한다[4]. 미국에서도 질병통제예방센터에 따르면 매년 약 250,000건의 혈류감염 사례가 보고되고 있다[5]. 카테터 관련 혈류감염은 환자의 입원 기간과 의료비용 및 사망률을 증가시키는 등 환자의 건강을 위협하는 중요한 문제이므로 혈류감염을 예방하는 것이 중요하다[6].

혈류감염을 효과적으로 예방하고 관리하기 위해 국내에서는 2006년부터 의료관련감염 감시체계(Korean National Healthcare-associated Infections Surveillance System, KONIS)를 운영하고 있다. KONIS는 진단기준에 따라 참여병원의 혈류감염 발생을 감시하고, 감시 자료를 등록하여 전체 혈류감염 발생률을 공유할 수 있는 네트워크이다[1]. KONIS 혈류감염 감시체계는 종합병원을 대상으로 적용할 때 혈류감염의 발생 규모를 파악하기에 몇 가지 제한점이 있다. 첫째, 일반병동에 대한 혈류감염 감시는 월 10건 이상의 중심정맥관 삽입, 유지가 이루어지는 병동에 대해 제한적으로 중심정맥관 관련 혈류감염(central-line associated bloodstream infection, CLABSI)만을 포함하고 있다[7]. 둘째, CLABSI의 발생률은 중심정맥관 1,000 사용일 당 발생 건수로 산출되기 때문에 중심정맥관 사용일수가 지표에 큰 영향을 미친다[1]. 400병상 미만의 중소병원들은 400-700병상 규모의 병원에 비해 중심정맥관 기구사용비나 CLABSI 발생률 모두 유의미하게 낮게 나타났다[8]. 이러한 결과는 규모가 큰 종합병원들에 비해 중소병원이 혈류감염의 가장 중요한 위험요인인 중심정맥관의 사용비가 낮은 것에 기인된 것으로 해석할 수 있다. 2020년 7월부터 2021년 6월까지 KONIS에 보고된 병상 규모에 따른 CLABSI의 기구사용비 역시 900병상 이상일 때 0.72이었으나 300-499병상일 때 0.49, 200-299병상일 때 0.30으로 병상 규모가 작아짐에 따라 기구 사용비가 작아지는 것을 확인할 수 있었다[4]. 상급종합병원에 비해 중심정맥관 삽입 건수와 사용일수가 적은 종합병원 및 병원은 CLABSI 감시만으로는 혈류감염 발생의 전반적인 규모 파악이 어렵다.

이러한 혈류감염 감시의 제한점을 보완할 수 있는 새로운 감시체계로 병원에서 발생하는 모든 혈류감염을 감시할 수 있는 병원발생 혈류감염(hospital-onset bloodstream infection, HOBSI) 감시를 제시하였다[9]. HOBSI의 기준이 규정되지 않았기 때문에 선행연구[9]에서는 NHSN (National Healthcare Safety Network) LabID (Laboratory-identified)와 검사로 확인된 혈류감염(Laboratory-confirmed bloodstream infection, BSI-LCBI) 정의를 사용하여 진단하였다. HOBSI는 중심정맥카테터뿐만 아니라 말초정맥카테터와 관련된 혈류감염까지 모두 포함하여 감시하고, 중환자실 및 병동에 입원한 환자 전체를 대상으로 감시할 수 있다는 장점이 있다. NHSN LabID 기준은 진단검사결과만으로 혈류감염을 정의하기 때문에 혈류감염 발생 전체를 확인할 수 있다. 그에 비해 BSI-LCBI의 경우 다른 부위와 관련된 이차 감염을 제외하므로 침습적인 카테터 삽입과 직접적인 연관성을 가진 예방 가능한 의료관련 혈류감염의 규모를 확인할 수 있다. HOBSI 감시를 적용한 선행연구[10]에 따르면 HOBSI 감시를 도입한 이후 전반적인 의료관련감염이 감소하였다. 이 결과는 HOBSI 감시를 활용하는 것이 병원에서 발생한 모든 혈류감염 감시에 유용하며, 예방 가능한 혈류감염에 더 민감한 지표가 될 수 있다는 것을 보여준다. 이에 종합병원에서 발생하는 혈류감염 발생의 규모를 정확히 파악하기 위해서 HOBSI 감시를 적용해 볼 수 있다.

한편, 상급종합병원에서 HOBSI 감시를 적용한 선행 연구[9]에서는 연구 기간 동안 발생한 혈류감염 사례의 48%가 말초정맥카테터만을 보유하고 있었다고 보고하였다. 또한, 혈류감염의 원인 미생물 중 황색포도알균(*Staphylococcus aureus*)과 혈장응고효소 음성 포도알균(Coagulase-negative staphylococci)이 가장 많이 분리되었다고 하였다. 이는 말초정맥카테터 관련 혈류감염(peripheral venous catheter-associated bloodstream infection, PVC-BSI)의 원인 미생물 조사[11,12]와 일치하는 결과이며, PVC-BSI 감시 및 중재의 필요성을 나타낸다. 하지만 현재 카테터 관련 혈류감염에 대한 대부분의 감시와 연구는 중심정맥카테터에 초점을 맞추고 있으며 PVC-BSI에 대한 연구는 매우 부족하다. 말초정맥카테터의 사용은 계속해서 증가하고 있다. 선행연구에서는 10년간 말초정맥카테터를 사용한 입원 환자가 3배가량 증가하였다고 보고하였다[13]. 입원하는 동안 대다수 환자가 말초정맥카테터를 보유하지만[14], 실제로 말초정맥카테터의 4%-28%는 치료에 사용되지 않고 카테터 보유 일수의 20%는 불필요한 것으로 추정한다[14-16]. 이처럼 말초정맥카테터는 혈류감염의 위험이 낮다고 간주되기 때문에 무분별하게 사용되어 PVC-BSI 발생의 원인이 되기도 한다[17]. 선행연구[18]에 따르면 PVC-BSI의 발생률은 0.18%로, 이는 병원 내 혈류감염의 6.3%, 카테터 관련 혈류감염의 23%를 차지한다. 최근 연구에서는 혈류감염 사례의 절반 정도가 말초정맥카테터만을 보유하고 있었고[9], 2020년에 비해 2021~2022년 동안 PVC-BSI가 증가하는 경향성도 보고된 바 있다[19]. 이처럼 말초정맥카테터는 다른 혈관카테터와 비교하여 사용량이 월등히 많기 때문에 혈관카테터 관련 감염에 미치는 영향을 무시할 수 없다. 하지만 KONIS의 혈류감염 감시체계에서 말초정맥카테터는 감시대상에 포함되지 않아서 PVC-BSI에 대한 결과는 알 수 없다[20,21]. 말초정맥카테터를 보유한 환자 중 혈류감염 진단기준을 만족하는 PVC-BSI의 역학적 특성과 결과를 알아볼 필요가 있다.

따라서 본 연구의 목적은 HOBSI 감시를 통해 중심정맥카테터와 말초정맥카테터를 포함한 모든 혈류감염 발생률을 파악하고, 기존 감시체계의 감시대상에 포함되지 않는 PVC-BSI의 위험요인 및 결과를 분석함으로써 종합병원 내 혈류감염 현황과 역학적 특성을 파악하는 것이다.

### 2. 연구 목적

본 연구에서는 종합병원의 HOBSI 발생률과 역학적 특성을 파악하고, PVC-BSI의 위험요인과 결과를 확인하고자 한다. 구체적인 목적은 다음과 같다.

1) HOBSI 진단기준에 따른 종합병원의 혈류감염 발생률과 역학적 특성을 파악한다.

2) PVC-BSI 발생 위험요인과 혈류감염의 임상 결과를 분석하고 CLABSI의 임상 결과와 비교한다.

## 연구 방법

### 1. 연구 설계

본 연구는 종합병원의 HOBSI 발생률과 역학적 특성을 파악하고, 말초정맥카테터 삽입 환자를 대상으로 혈류감염 발생군과 비발생군을 선정하여 PVC-BSI의 위험요인 및 결과를 분석하는 후향적 사례-대조군 연구이다.

### 2. 연구 대상

378병상 규모의 종합병원인 좋은삼선병원에서 2021년 8월 1일부터 2023년 7월 31일 사이에 중심정맥카테터와 말초정맥카테터를 모두 포함하는 혈관카테터를 연속적으로 3일 이상 보유한 재원 환자 중 만 18세 이상의 환자 21,708명을 대상으로 하였다. 전체 환자를 대상으로 HOBSI의 발생률과 역학적 특성을 파악하였다.

PVC-BSI는 말초정맥카테터 보유 환자만을 대상으로 조사하였다. 대상자의 처방 기록과 투약 기록을 검토하여 주사 처방 및 투약 기록이 연속적으로 3일 이상 있는 경우 말초정맥카테터를 연속적으로 3일 이상 보유한 것으로 하였다.

사례군은 전체 대상자 중 말초정맥카테터를 연속적으로 3일 이상 보유한 재원 환자 중 KONIS의 혈류감염 기준에 따라 혈류감염이 발생한 환자 31명이다. 중심정맥카테터를 보유하고 있고 KONIS의 CLABSI 기준을 만족하는 58명은 사례 대조군 분석에서 제외하였고, PVC-BSI와의 임상 결과를 비교하기 위한 CLABSI 발생군으로만 포함되었다.

대조군은 혈류감염이 발생하지 않은 환자로 성별 및 연령과 주요 혈류감염 위험요인으로 규명된 악성종양 유무, 면역억제제 복용 여부, 호중구 감소 여부, 카테터 보유력(말초 및 중심정맥카테터 보유 여부)으로 성향점수 매칭(Propensity Score Matching) 방법을 통해 선정하였다. 이때 사례군에 대한 대조군 매칭 비율은 통계적 검정력을 확보하고, 발생할 수 있는 편향을 최소화하기 위해 1:3으로 하여[22] 93명의 대조군을 선정하였다. 성향점수 매칭은 대상자 선정 시 선택 편의를 교정하기 위해 교란 요인으로 작용할 수 있는 변수를 공변량으로 투입하여 매칭하는 방법이다[23]. 본 연구에서는 혈류감염 발생에 영향을 미칠 것으로 예상되는 성별 및 연령과 주요 혈류감염 위험요인을 공변량으로 투입하여 성향점수를 추정하였다. 그리고 사례군의 추정된 성향점수와 가장 유사한 성향점수를 가진 대조군을 매칭하였다.

### 3. 연구 도구

HOBSI를 규명하기 위해 선행연구에서 적용한 NHSN LabID 정의[24]와 KONIS Manual의 혈류감염 기준[1]에 따라 사례를 정의하고 각각 HOBSI-1, HOBSI-2라고 하였다. HOBSI-1은 증상과 상관없이 혈액 배양검사 결과만으로 혈류감염 여부를 진단하며, HOBSI-2는 혈액 배양검사 결과와 증상을 모두 고려하여 진단을 하며 이차혈류감염을 제외한다. CLABSI는 HOBSI-2를 만족하는 환자 중 2일을 초과하여 중심정맥카테터를 가지고 있었고 감염발생일 또는 그 전날 중심정맥카테터를 가지고 있었던 경우로 정의하였다. PVC-BSI는 HOBSI-2를 만족하는 환자 중 2일을 초과하여 말초정맥카테터를 가지고 있었고 감염발생일 또는 그 전날 말초정맥카테터를 가지고 있었던 경우로 정의하였다(Table 1).

KONIS Manual[1]의 감염환자 기록지와 대한의료관련감염관리학회의 의료관련감염관리[20]를 참고하여 연구자가 증례 기록서를 개발하였다. 대상자 관련 요인, 치료 관련 요인과 혈류감염의 역학적 특성, 임상 결과로 구성하였다. 대상자 관련 요인으로는 연령, 성별, 재원일수, 진료과, 입원 경로, 기저질환, 면역억제제 복용 여부, 호중구 감소 여부를 포함하였다. 치료 관련 요인으로는 수술 여부, 침습적 기구 사용 및 침습적 처치 여부, 항생제 사용 여부, TPN (total parenteral nutrition) 투여 여부, 정맥내지방 투여 여부, 수혈 여부를 포함하였다. 혈류감염 역학적 특성으로는 혈류감염 발생 여부, 검체 접수 일자, 감염발생일, 감염 발생 당시 입원 장소, 원인 미생물과 내성균을 포함하였다. 임상 결과로는 대상자의 입원 기간 내 사망 여부를 의무기록으로 확인하였다.

### 4. 자료 수집

2021년 8월 1일부터 2023년 7월 31일 사이에 혈관카테터를 연속적으로 3일 이상 보유한 재원 환자 중 만 18세 이상의 환자를 대상으로 의무기록을 확인하여 수집하였다. 좋은강안병원 임상연구심의윤리위원회의 승인을 받은 후 연구기관에서 허가받은 ID를 통해 해당 기간 입원한 환자의 목록을 확인하였다. 목록을 토대로 연구자가 의무기록에 접근하여 분석에 필요한 정보를 수집하였고, 수집된 데이터에는 대상자의 개인 정보를 포함하지 않고 ID를 부여하여 익명화하였다.

### 5. 자료 분석

수집된 자료의 통계 분석은 SPSS version 27.0 (IBM corp., Armonk, NY, USA)을 이용하였다. HOBSI의 발생률은 1,000 환자일수 당 혈류감염 발생 건수(HOBSI-1, HOBSI-2, CLABSI)로 분석하였다. HOBSI의 역학적 특성에 대한 자료는 빈도, 백분율을 이용하여 분석하였다. PVC-BSI 발생에 영향을 주는 위험요인과 혈류감염의 임상 결과는 χ^2^-test와 independent t-test로 분석하였다. 그리고 여러 위험요인 변수를 동시에 고려한 다변량 분석을 위해 로지스틱 회귀분석을 실시하여 보정된 odds ratio (OR)와 95% confidence interval (CI)을 계산하였다.

### 6. 윤리적 고려

본 연구는 좋은강안병원의 임상연구심의윤리위원회의 승인을 받았다(IRB No. GGAH 2023-10). 수집된 자료는 대상자를 식별할 수 있는 개인 정보를 모두 제외하고 암호화된 문서로 보관하였으며 패스워드를 설정하여 연구자 이외의 타인은 접근할 수 없도록 하였다.

## 연구 결과

### 1. HOBSI의 발생률 및 역학적 특성

혈류감염 진단기준은 Table 1과 같다. 증상과 상관없이 혈액 배양검사 결과만으로 진단하는 HOBSI-1을 만족하는 혈류감염은 총 136건이었으며 발생률은 1,000 환자일수 당 0.61이었다. HOBSI-1과 다르게 이차혈류감염은 제외되는 HOBSI-2를 만족하는 혈류감염은 총 89건이었으며 발생률은 1,000 환자일수 당 0.40이었다. HOBSI-2를 만족하는 환자 중 중심정맥카테터 보유 기준을 만족하는 CLABSI의 발생률은 1,000 환자일수 당 0.25이었다(Appendix 1).

진단기준별 혈류감염의 역학적 특성은 Table 2와 같다. 말초정맥카테터만을 보유하고 있는 사례는 HOBSI-1 136건 중 39건(28.7%)이었고, HOBSI-2 89건 중 30건(33.7%)이었다. 중심정맥카테터만을 보유하고 있는 사례는 HOBSI-1 136건 중 59건, HOBSI-2 89건 중 24건이었으며 말초정맥카테터와 중심정맥카테터를 동시에 보유하고 있는 사례는 HOBSI-1 136건 중 37건, HOBSI-2 89건 중 34건이었다. 기타(케모포트)는 1건이었다. 혈류감염이 발생하기까지의 기간은 HOBSI-1, HOBSI-2는 평균 30일, 중앙값 22일로 큰 차이가 없었으며 CLABSI는 평균 39일, 중앙값 32일이었다. HOBSI-1의 혈류감염 발생 당시 입원 장소는 일반병동이 96건(70.6%), 중환자실이 40건(29.4%)이었으며 HOBSI-2는 일반병동이 71건(79.8%), 중환자실이 18건(20.2%)이었다. CLABSI의 경우 일반병동에서 42건(72.4%), 중환자실에서 16건(27.6%)이 발생하였다. 원인 미생물은 HOBSI-1의 경우 Coagulase negative staphylococci가 26건(19.1%)으로 가장 많았고 그다음으로 *Staphylococcus aureus* 17건(12.5%), *Escherichia coli* 16건(11.8%), *Klebsiella* species 14건(10.3%) 순이었다. HOBSI-2는 *Escherichia coli*가 12건(13.5%)으로 가장 많았으며, *Staphylococcus aureus* 11건(12.4%), *Acinetobacter baumannii* 11건(12.4%), *Enterobacter cloacae* 11건(12.4%), Others 10건(11.2%), *Klebsiella* species 9건(10.1%), Coagulase negative staphylococci 7건(7.9%), *Pseudomonas aeruginosa* 6건(6.7%), *Candida* species 6건(6.7%), *Stenotrophomonas maltophilia* 3건(3.4%), *Enterococcus faecalis* 2건(2.2%), *Enterococcus faecium* 2건(2.2%), *Serratia marcescens* 1건(1.1%)으로 나타났다.

### 2. PVC-BSI 발생 위험요인

#### 1) 대상자 및 치료 관련 위험요인

혈류감염이 발생한 환자의 대상자 및 치료 관련 위험요인은 Table 3과 같다. PVC-BSI가 발생한 환자는 31명이었고 남자가 19명(61.3%), 여자가 12명(38.7%)이었다. 혈류감염이 발생하지 않은 대조군에서 남자는 54명(58.1%), 여자가 39명(41.9%)이었다. 평균 재원일수는 사례군이 33.94 ± 27.59일, 대조군이 19.53 ± 20.01일이었으며 통계적으로 유의한 차이가 있었다(t = −2.68, *p* = .010). 사례군에서 기저질환이 있는 환자는 21명(67.7%)이었고 대조군에서 68명(73.1%)으로 통계적으로 유의한 차이는 없었다. 기저질환의 종류별로 살펴보면 간경화가 있는 환자가 사례군에서 12.9%, 대조군에서 1.1%로 통계적으로 유의한 차이가 있었다(χ² = 5.63, *p* = .018). 그 외에 당뇨, 고혈압 악성종양, 만성 신질환, 만성 폐쇄성 폐질환, 천식은 유의한 차이가 없었다. 수술 경험이 있는 환자는 사례군에서 10명(32.3%), 대조군에서 36명(38.7%)으로 통계적으로 유의한 차이가 없었다. 침습적 기구를 사용한 환자는 사례군에서 12명(38.7%), 대조군에서 32명(34.4%)이었으며 침습적 기구의 종류별로 살펴보면 인공호흡기, 유치도뇨관, 투석도관, 비위관, 배액관 모두 유의한 차이가 없었다. 또한, 침습적 처치를 시행한 환자는 사례군에서 4명(12.9%), 대조군에서 10명(10.8%)으로 통계적으로 유의한 차이가 없었으며, 종류별로 살펴보았을 때도 혈액투석, 심혈관 시술에서 유의한 차이가 없었다. 항생제를 사용한 환자는 사례군에서 15명(48.4%), 대조군에서 52명(55.9%)이었고 두 군 간에 통계적으로 유의한 차이는 없었다. 수혈한 환자는 사례군에서 5명(16.1%), 대조군에서 8명(8.6%)으로 유의한 차이가 없었으며 TPN과 정맥내지방 투여 여부 역시 유의한 차이가 없었다.

#### 2) PVC-BSI 발생 위험요인에 대한 다변량 분석

PVC-BSI 발생 위험요인에 대한 단변량 분석에서 유의한 변수들을 선택하여 다중로지스틱 회귀분석 시행결과 PVC-BSI에 유의한 위험요인은 재원일수(OR = 1.03, 95% CI = 1.01-1.05), 기저질환 중 간경화(OR = 14.76, 95% CI = 1.57-139.04)로 나타났다. PVC-BSI는 재원일수가 하루 길어질 때마다 1.03배 높은 것으로 나타났고, 간경화가 있는 환자의 경우 14.76배 높은 것으로 나타났다(Table 4).

### 3. PVC-BSI의 임상 결과

PVC-BSI가 발생한 환자 31명 중 입원 중 사망한 환자는 6명(19.4%)으로 혈류감염이 발생하지 않은 환자에 비해 생존한 상태로 퇴원하는 비율이 매우 유의하게 낮았다(*p* < .001). PVC-BSI가 발생한 환자는 혈류감염이 발생하지 않은 환자보다 사망에 대한 위험도가 약 22.08배 증가하였다. 같은 기간 동안 CLABSI의 경우 사례군 58명 중 퇴원 시점에 사망한 환자는 16명(27.6%), 대조군에서 16명(9.2%)으로 사례군에서 퇴원 시 사망한 경우가 3.76배 높게 나타났다(Table 5).

## 논의

본 연구는 종합병원의 HOBSI 감시를 통해 혈류감염 발생률을 파악하고, PVC-BSI의 위험요인 및 결과를 분석함으로써 종합병원 내 혈류감염 현황과 역학적 특성을 파악하고자 시행되었다. 진단기준별 혈류감염 발생률을 비교한 결과, HOBSI-2 발생률이 기존의 혈류감염 감시체계인 CLABSI 발생률보다 1.6배 높은 것으로 나타났다. 이는 종합병원에서 HOBSI 감시를 적용하는 것이 예방 가능한 혈류감염 감시의 더 민감한 지표가 될 수 있다는 것을 뜻한다. 이러한 혈류감염은 일반병동에서 약 70% 이상 발생하고 있어 중환자실만을 의료관련감염의 주요 감시대상으로 포함하는 것은 의료관련감염의 규모를 확인하는데 충분하지 않음을 시사한다. 또한, PVC-BSI 사례의 사망에 대한 OR이 22.08로 높게 나타나 HOBSI 진단기준을 적용하는 것이 최적의 감염관리 실무 및 환자 안전을 보장하기 위해 고려해볼 가치가 있음을 뒷받침한다.

진단기준별 혈류감염의 발생률은 HOBSI-1 적용 시 1,000 환자일수당 0.61, HOBSI-2 적용 시 1,000 환자일수당 0.40, CLABSI는 1,000 환자일수당 0.25였다. Gurney 등[9]의 선행연구에서 HOBSI-2의 발생률이 CLABSI보다 2배 높았으며, 본 연구에서도 HOBSI-2의 발생률이 CLABSI보다 1.6배 높았다. HOBSI의 발생률이 CLABSI의 발생률보다 높게 나타난 것은 현재 혈류감염 감시체계에서 제외되고 있는 혈류감염 사례가 있다는 것을 의미한다. CLABSI 감시 기준의 단점으로 민감도와 특이도가 제한적이며 이에 따라 CLABSI가 과소보고 또는 과다보고 될 수 있다는 것이 지적되고 있기 때문에 더 민감한 혈류감염 감시 지표로 HOBSI를 선택하는 것을 고려할 수 있다[25]. 5년 동안 HOBSI와 CLABSI를 비교한 선행연구[26]에 따르면 시간이 지남에 따라 HOBSI의 발생률은 증가했지만 CLABSI 발생률은 증가하지 않았다. 즉, CLABSI 감시만으로는 혈류감염 발생 규모를 파악하기에 충분하지 않을 수 있기 때문에 이런 측면에서 HOBSI 감시체계가 더 적합한 진단기준이 될 수 있다. 본 연구에서 HOBSI-1은 CLABSI보다 2배 이상 높은 발생률을 보였으나, 임상 증상과 무관하게 혈액배양검사 결과를 기반으로 감시하기 때문에 이차 혈류감염을 배제할 수 없다. HOBSI-1은 혈류감염 감시를 위해 소요되는 시간과 노력이 비교적 적지만, 기존의 혈류감염 감시 자료와 직접 비교하기 어렵고 중재 계획 수립 시 이차 혈류감염을 고려해야 한다. 반면 HOBSI-2는 기존의 KONIS의 혈류감염 감시체계 기준을 사용하며, CLABSI에 비해 전반적인 감염관리 효과에 대한 총체적인 평가를 제공할 수 있다. 국외의 설문 조사에 따르면, 감염관리 직원은 의료관련감염 감시에만 36%의 시간을 할애하고 있다고 한다[27]. 감염관리를 위한 인력과 자원이 한정적이기 때문에 기존의 감시체계는 위험도가 높은 부서를 우선적으로 고려하였다. 하지만 최근 인공지능(artificial intelligence, AI)을 활용한 의료관련감염 감시의 효과를 평가하는 연구가 이루어지고 있으며, 감염 사례 발견 및 역학 조사 등에서 상당한 효과가 있다는 것을 확인하였다[28]. AI 기반 감시 도구는 방대한 양의 자료를 처리하고 분석할 수 있으며 노동집약적인 과정을 간소화하여 시간을 절약할 수 있다. 또한, 18개월 동안 AI 알고리즘을 적용하여 의료관련감염 감시를 시행한 선행연구[29]에서 의료관련감염이 발생한 73명의 환자 중 67명을 정확하게 감지하였다. 이처럼 AI 기반 감시 도구를 추가로 활용하는 것도 인력과 자원이 한정된 병원에서 민감도가 높은 HOBSI 감시를 도입하기 위한 좋은 방법이 될 수 있다.

본 연구에서 혈류감염이 발생한 위치는 일반병동에서 발생한 비율이 HOBSI-1, HOBSI-2, CLABSI 각각 70.6%, 79.8%, 72.4%로 중환자실보다 훨씬 높게 나타났다. 이는 PVC-BSI의 대부분이 일반병동에서 발생했다는 선행연구와 일치하며[30], CLABSI의 약 40%~80%가 일반병동에서 발생하였다는 연구 결과와도 일치한다[31,32]. 2년간 HOBSI를 감시한 선행연구[33]에서도 HOBSI의 67.1%가 일반병동에서 발생했다. 대부분의 감시와 중재는 중환자실의 CLABSI에 초점을 맞추고 있으나, 최근 감시대상과 범위를 확대할 필요성이 있다는 인식이 확산되고 있다. 본 연구 결과 역시 혈류감염 감시대상을 일반병동까지 확대하여 병원 전체를 감시했을 때 70% 이상의 혈류감염이 일반병동에서 발생했으며, 이는 중환자실뿐만 아니라 일반병동에서도 혈류감염의 감시가 필요하다는 것을 알 수 있다. 중환자실은 환자의 중증도가 높으며 삽입기구의 사용이 빈번하여 혈류감염에 대한 감시 및 감염관리 중재의 우선순위가 높은 부서이다[20]. 일반병동은 중환자실에 비해서 환자의 중증도는 낮을 수 있지만, 간호사당 환자 수가 많기 때문에 혈류감염 발생 사례의 발견 및 감염관리 적용이 지연될 수 있다[34]. 간호 인력 확보수준과 의료관련감염 발생의 연관성을 조사한 연구에서 간호 인력이 부족할수록 의료관련감염이 증가하였으며, 의료관련감염 감시 간호 인력이 추가되었을 때 혈류감염이 감소하였다[35]. 이러한 결과를 토대로 혈류감염 감시대상을 일반병동까지 확대한다면 전반적인 의료관련감염이 감소하는 효과를 기대할 수 있다.

PVC-BSI가 발생한 환자들의 사망에 대한 OR은 22.08로 높게 나타났다. PVC-BSI가 발생한 환자들의 사망률은 19.4%였으며 선행 연구[30,36,37]에서도 PVC-BSI가 발생한 환자들의 사망률이 12%~29%로 나타난 것과 유사한 결과를 보였다. PVC-BSI가 발생한 환자들의 사망률은 혈류감염이 발생하지 않은 환자보다 22.08배 높았으며, 이는 CLABSI의 예후보다 더 나쁜 결과로 나타났다. Tsuboi 등[38]의 연구에서는 7일 사망률이 말초정맥카테터와 중심정맥카테터 관련 혈류감염에서 비슷했으며, 30일 사망률은 CLABSI가 더 높게 나타났다는 결과와 차이가 있었다. 하지만 CLABSI와 CLABSI를 제외한 HOBSI의 사망률을 분석한 선행연구[26]에서, 단변량 분석 결과 두 군 사이에 유의미한 차이가 없었으나 다변량 분석에서 질병의 중증도를 예측하는 공변량을 조정했을 때, CLABSI보다 CLABSI를 제외한 HOBSI의 사망률이 높게 나타났다. 연구마다 연구 방법 및 대상자에서 차이가 있고 본 연구가 후향적 사례-대조군 연구이므로 인과관계 해석 시 제한적이지만, 이상의 결과는 CLABSI가 아닌 PVC-BSI 역시 사망의 위험성을 주요하게 다루어야 한다는 점을 시사한다.

한편, 본 연구는 기존 감시체계의 감시대상에 포함되지 않는 PVC-BSI의 위험요인을 규명하고자 하였으며, 다변량 분석을 시행한 결과 재원일수와 기저질환 중 간경화가 유의미한 요인으로 나타났다. 재원일수가 하루 길어질 때마다 PVC-BSI 발생 확률이 1.03배 증가하였으며, Yoo 등[39]의 연구에서도 재원 기간이 혈류감염에 영향을 미치는 중요한 요인이었다. 따라서 말초정맥카테터의 불필요한 사용을 줄이고, 신속하게 제거할 수 있도록 중재할 필요가 있다. 또한, 본 연구에서 간경화를 가진 환자가 PVC-BSI 발생 가능성이 유의미하게 높은 것으로 나타났는데, 이는 간경화가 아닌 환자에 비해 간경화 환자에서 혈류감염 발생위험이 10배로 나타난 선행연구결과와 일치한다[40]. 간경화 환자들은 여러 가지 생리적, 면역학적 변화로 인해서 감염 발생에 취약하다. 합병증인 비장비대로 인한 백혈구 감소, 간 기능 저하로 인한 면역 기능장애가 발생하고, 문맥 고혈압으로 인한 세균 과증식 촉진, 영양 불량 상태 등으로 인해 세균감염에 취약해진다[41,42]. 감염은 간경화 환자들에게 간부전을 야기하는 촉발요인이 되고, 사망위험을 증가시킨다[43]. The Model for End-stage Liver Disease (MELD) 점수가 간경화 환자의 혈류감염 결과에 유의미한 요인으로 나타났으므로[44], 특히 MELD 점수가 높거나 악화되는 환자의 경우 혈류감염 발생의 고위험군으로 간주하여 선제적인 감염관리를 적용해야 할 것이다.

본 연구에는 몇 가지 제한점이 있다. 첫째, 일개 종합병원의 단일기관 연구이므로 다른 병원으로의 일반화에는 한계가 있다. 둘째, 후향적으로 의무기록을 검토하여 자료를 수집하였기 때문에 감염에 영향을 미치는 외생변수인 PVC-BSI 관리 행위 양상, 간호 인력 수준, 계절적 요인, 손위생 수행률, 카테터 삽입 위치, 카테터 유치 기간, 카테터 교체 주기 등을 충분히 고려하지 못할 가능성도 존재한다. 또한, 연구기관에서 입원 환자 전체를 대상으로 중증도를 확인할 수 있는 기록이 없어 환자의 중증도를 고려하지 못하였다. 셋째, 말초정맥카테터 기구일수 대신 환자일수를 사용하여 HOBSI 발생률을 산출하였으므로 정확한 HOBSI 발생률을 파악하고 CLABSI 발생률과 비교하기 위해서는 말초정맥카테터의 기구일수를 사용하여 혈류감염 발생률을 산출하고, 그 차이를 살펴볼 필요가 있다. 마지막으로, 본 연구에서 사용한 혈류감염의 진단기준은 혈류감염 감시를 위한 목적이므로 임상적인 감염을 의미하는 것은 아니다. 이러한 제한점에도 불구하고 본 연구는 새로운 혈류감염 감시체계인 HOBSI 감시를 통해 중심정맥카테터와 말초정맥카테터 관련 혈류감염을 모두 포함한 종합병원의 혈류감염 실태를 파악하였으며, 국내에서 드물게 연구되었던 주제인 PVC-BSI의 위험요인과 결과를 분석한 점에서 의의가 있다. 이를 통해 종합병원에서 혈류감염을 예방하기 위한 새로운 중재 방안을 모색할 때, 더 민감한 지표가 될 수 있는 감시체계를 제시했다는 점에서 의미가 있다고 사료된다.

## 결론

본 연구에서 진단기준별 혈류감염 발생률을 확인한 결과, HOBSI-1, HOBSI-2 발생률이 CLABSI 발생률보다 높게 나타났고 중환자실보다 일반병동에서 발생하는 혈류감염의 비중이 높았다. 이를 통해 현재 혈류감염 감시체계에서는 종합병원에서 파악하기 어려운 혈류감염이 있다는 것을 확인하였다. 따라서 상급종합병원에 비해 중심정맥카테터 사용이 적은 종합병원에서는 혈류감염 예방을 위해 기존의 감시체계보다 더 민감한 지표인 HOBSI 감시체계로 혈류감염을 감시하는 것을 고려할 수 있다.

본 연구는 진단기준별 혈류감염 발생률을 통해 종합병원에서 발생하는 혈류감염 발생 규모를 정확히 파악하고 PVC-BSI의 위험요인 및 결과를 분석함으로써 종합병원 내 혈류감염의 역학적 특성을 살펴보았으며, 혈류감염 감시를 위한 더 민감한 지표를 선택하는 것에 기초자료를 제공했다는 점에서 의의가 있다. 이를 바탕으로 다양한 규모의 병원에서의 HOBSI 감시가 혈류감염 발생에 미치는 영향과 PVC-BSI의 위험요인 규명에 대한 반복연구를 제안한다.

## Figures and Tables

**Table 1.** Criteria and Case Definitions Used to Classify BSIs

BSI	Definitions
HOBSI-1 (NHSN LabID)	☐ Positive blood culture from blood samples of patients hospitalized for more than 3 calendar days
	☐ Patients with a common commensal from only one blood culture are excluded.
HOBSI-2 (BSI-LCBI)	At least one of the following diagnostic criteria (1 or 2) must be met:
	
	[Diagnostic Criterion 1]
	☐ Pathogen is identified from one or more blood specimens obtained for clinical diagnosis or treatment purposes, and the organisms isolated from blood samples are not related to infections at other body sites.
	
	[Diagnostic Criterion 2]
	☐ The patient has at least one of the following symptoms or signs: fever (> 38°C), chills, or hypotension
	
	**AND**
	
	☐ Skin commensal organisms are identified from two or more independently collected blood specimens, with the organisms isolated from blood samples not related to infections at other body sites.
CLABSI (BSI-central line)	☐ Cases are confirmed to have HOBSI-2
	
	**AND**
	
	☐ The patient has an eligible central venous catheter in place for more than 2 calendar days
	
	**AND**
	
	☐ The eligible central line is present on the day of event (infection) or the day before
PVC-BSI	☐ Cases are confirmed to have HOBSI-2

	**AND**

	☐ The patient has only peripheral venous catheters in place for more than 2 calendar days

	**AND**

	☐ The eligible peripheral line is present on the day of event (infection) or the day before

BSI = Bloodstream infection; HOBSI = Hospital-onset bloodstream infection; NHSN = National Healthcare Safety Network; LabID = Laboratory-identified; LCBI = Laboratory-confirmed bloodstream infection; CLABSI = Central line associated bloodstream infection; PVC-BSI = Peripheral venous catheter-associated bloodstream infection.

**Table 2.** General Epidemiology of HOBSIs and CLABSIs

Variables	HOBSI-1 (n = 136)	HOBSI-2 (n = 89)	CLABSI (n = 58)
	n (%)	n (%)	n (%)
Only peripheral line	39 (28.7)	30 (33.7)	0
Days to infection	3~150	3~150	5~150
M ± SD	29.79 ± 28.54	29.79 ± 28.17	39.43 ± 29.64
Median (IQR)	22 (8~41)	22 (9~41)	32 (17~49)
Attributable unit			
Intensive care unit	40 (29.4)	18 (20.2)	16 (27.6)
General ward	96 (70.6)	71 (79.8)	42 (72.4)
Isolated microorganism^†^			
Gram-positive cocci			
*Staphylococcus aureus*	17 (12.5)	11 (12.4)	4 (6.9)
Coagulase-negative staphylococci	26 (19.1)	7 (7.9)	4 (6.9)
*Enterococcus faecium*	6 (4.4)	2 (2.2)	2 (3.4)
*Enterococcus faecalis*	4 (2.9)	2 (2.2)	2 (3.4)
Gram-negative bacilli			
*Escherichia coli*	16 (11.8)	12 (13.5)	3 (5.2)
*Acinetobacter baumannii*	12 (8.8)	11 (12.4)	11 (19.0)
*Enterobacter cloacae*	11 (8.1)	11 (12.4)	7 (12.1)
*Klebsiella* species	14 (10.3)	9 (10.1)	8 (13.8)
*Pseudomonas aeruginosa*	7 (5.2)	6 (6.7)	3 (5.2)
*Stenotrophomonas maltophilia*	4 (2.9)	3 (3.4)	3 (5.2)
*Serratia marcescens*	1 (0.7)	1 (1.1)	1 (1.7)
Other			
*Candida* species	8 (5.9)	6 (6.7)	6 (10.3)
Other	10 (7.4)	10 (11.2)	6 (10.3)

HOBSI = Hospital-onset bloodstream infection; CLABSI = Central line-associated bloodstream infection; M = Mean; SD = Standard deviation; IQR = Interquartile range. ^†^Multiple responses.

**Table 3.** Comparison of Characteristics Between Case and Control Groups for Peripheral Venous Catheter-associated Bloodstream Infections

Variables	Case (n = 31)	Control (n = 93)	t/χ²	*p*
	n (%) or M ± SD	n (%) or M ± SD		
Sex				
Men	19 (61.3)	54 (58.1)	0.01	.916
Women	12 (38.7)	39 (41.9)		
Age (yr)	69.94 ± 14.51	70.10 ± 14.00	0.05	.957
Duration of admission (day)	33.94 ± 27.59	19.53 ± 20.01	-2.68	.010
Route of admission				
Emergency room	13 (41.9)	44 (47.3)	0.10	.755
Outpatient department	18 (58.1)	49 (52.7)		
Underlying diseases				
No	10 (32.3)	25 (26.9)	0.12	.730
Yes^†^	21 (67.7)	68 (73.1)		
Diabetes mellitus	4 (12.9)	30 (32.3)	3.46	.063
Hypertension	14 (45.2)	52 (55.9)	0.69	.406
Cancer	4 (12.9)	13 (14.0)	0.00	>.999
Chronic kidney disease	1 (3.2)	5 (5.4)	0.00	>.999
COPD	0 (0.0)	2 (2.2)	0.00	>.999
Liver cirrhosis	4 (12.9)	1 (1.1)	5.63	.018
Asthma	0 (0.0)	3 (3.2)	0.11	.736
Others	0 (0.0)	3 (3.2)	0.00	>.999
Operation				
No	21 (67.7)	57 (61.3)	0.18	.668
Yes	10 (32.3)	36 (38.7)		
Invasive device				
No	19 (61.3)	61 (65.6)	0.05	.828
Yes^†^	12 (38.7)	32 (34.4)		
Ventilator	1 (3.2)	0 (0.0)	0.34	.562
Urinary indwelling catheter	10 (32.3)	24 (25.8)	0.22	.642
Hemodialysis catheter	1 (3.2)	2 (2.2)	0.00	> .999
Tracheostomy	0 (0.0)	0 (0.0)	-	-
Levin tube	3 (9.7)	5 (5.4)	0.18	.673
Drainage tube	3 (9.7)	13 (14.0)	0.10	.757
Others	0 (0.0)	0 (0.0)	-	-
Invasive procedure				
No	27 (87.1)	83 (89.2)	0.00	> .999
Yes^†^	4 (12.9)	10 (10.8)		
Hemodialysis	1 (3.2)	3 (3.2)	0.00	> .999
Cardiovascular intervention	3 (9.7)	10 (10.8)	0.00	> .999
Antibiotics				
No	16 (51.6)	41 (44.1)	0.27	.603
Yes^†^	15 (48.4)	52 (55.9)		
Aminoglycosides	0 (0.0)	2 (2.2)	0.00	> .999
Cephalosporins	14 (45.2)	45 (48.4)	0.01	.917
Penicillin	0 (0.0)	10 (10.8)	2.32	.128
Glycopeptides	0 (0.0)	1 (1.1)	0.00	> .999
Carbapenems	1 (3.2)	0 (0.0)	0.34	.562
Tetracycline	1 (3.2)	1 (1.1)	0.00	> .999
Quinolones	5 (16.1)	12 (12.9)	0.02	.880
Others	1 (3.2)	0 (0.0)	0.34	.562
TPN				
No	26 (83.9)	81 (87.1)	0.02	.880
Yes	5 (16.1)	12 (12.9)		
Lipid				
No	29 (93.5)	89 (95.7)	0.00	> .999
Yes	2 (6.5)	4 (4.3)		
Transfusions				
No	26 (83.9)	85 (91.4)	0.72	.397
Yes^†^	5 (16.1)	8 (8.6)		
PRC	5 (16.1)	8 (8.6)	0.72	.397
PC	0 (0.0)	1 (1.1)	0.00	> .999
FFP	0 (0.0)	0 (0.0)	-	-

M = Mean; SD = Standard deviation; COPD = Chronic obstructive pulmonary disease; TPN = Total parenteral nutrition; PRC = Packed red blood cell; PC = Platelet concentrate; FFP = Fresh frozen plasma. ^†^Multiple responses.

**Table 4.** Multivariate Logistic Regression Analysis of Risk Factors for Peripheral Venous Catheter-associated Bloodstream Infections

Variables	*p*	OR	95% CI
Duration of admission (day)	.005	1.03	1.01-1.05
Liver cirrhosis	.019	14.76	1.57-139.04

OR = Odds ratio; CI = Confidence interval.

**Table 5.** Clinical Outcomes of Peripheral Venous Catheter-associated and Central line-associated BSIs

Variables			*p*	OR
Peripheral venous catheter-associated BSI	Case (n = 31)	Control (n = 93)		
Alive	25 (80.6)	92 (98.9)	< .001	22.08
Died	6 (19.4)	1 (1.1)		
Central line-associated BSI	Case (n = 58)	Control (n = 174)		
Alive	42 (72.4)	158 (90.8)	.001	3.76
Died	16 (27.6)	16 (9.2)		

Values are presented as n (%). BSI = Bloodstream infection; OR = Odds ratio.

## Appendix 1.

Incidence rates and incidence rate differences of hospital-onset bloodstream infection compared to central line-associated bloodstream infection for patients.

HOBSI = Hospital-onset bloodstream infection; CLABSI = Central line associated bloodstream infection.
